# Supplementary material for: RAB23 coordinates early osteogenesis by repressing FGF10-pERK1/2 and GLI1
Source: eLife. 2020 Jul 14;9:e55829. doi: 10.7554/eLife.55829 (PMC7423339; doi:10.7554/eLife.55829)
Supplement: Supplementary file 1. [file elife-55829-supp1.docx]

**Supplementary file 1:** List of 223 differentially expressed genes in *Rab23^-/-^* calvaria.

| **Orig_annotations** | **Gene symbol** | **Fold change** | **P-value** |
| --- | --- | --- | --- |
| ILMN_2608804 | *Ckm* | 8.987234836 | 0.030005 |
| ILMN_2691780 | *Myl3* | 8.505568492 | 0.019004 |
| ILMN_2918875 | *Sln* | 8.371168719 | 0.033031 |
| ILMN_1260428 | *Myl2* | 8.042741557 | 0.011855 |
| ILMN_2978038 | *Myh8* | 7.544301307 | 0.038747 |
| ILMN_2629581 | *Cox6a2* | 7.150349745 | 0.041803 |
| ILMN_2469018 | *Tnnt3* | 6.318070124 | 0.048545 |
| ILMN_2527465 | *Klhl41* | 4.847292532 | 0.030054 |
| ILMN_2768612 | *Atp1b4* | 4.526888561 | 0.028556 |
| ILMN_1234857 | *Myoz2* | 4.403513714 | 0.04736 |
| ILMN_2744469 | *Mybpc1* | 4.27668009 | 0.04371 |
| ILMN_1217077 | *Oc90* | 4.224583898 | 0.028488 |
| ILMN_2673358 | *Myom3* | 4.045694083 | 0.047408 |
| ILMN_2765759 | *Asb2* | 3.933384612 | 0.046329 |
| ILMN_2909248 | *Vgll2* | 3.877742688 | 0.031447 |
| ILMN_2694907 | *Myl1* | 3.863483833 | 0.012283 |
| ILMN_2706269 | *Hspb1* | 3.093664987 | 0.03288 |
| ILMN_1241866 | *Arpp21* | 2.886379287 | 0.046372 |
| ILMN_2605790 | *Lmod3* | 2.327600237 | 0.041551 |
| ILMN_2941790 | *Cldn6* | 2.321115852 | 0.045795 |
| ILMN_2727472 | *Isl1* | 2.257284651 | 0.022317 |
| ILMN_3118071 | *Pitx2* | 2.235170645 | 0.044963 |
| ILMN_2977056 | *Tnni1* | 2.152699247 | 0.044274 |
| ILMN_2596569 | *Ptges3l* | 2.093725281 | 0.038886 |
| ILMN_2789651 | *Csrp3* | 2.086183036 | 0.038728 |
| ILMN_2695143 | *Capn6* | 2.054524534 | 0.027253 |
| ILMN_1218266 | *Meis1* | 2.046154133 | 0.02327 |
| ILMN_2632665 | *Cav1* | 2.046132756 | 0.035748 |
| ILMN_3118584 | *Bex4* | 2.030798523 | 0.041515 |
| ILMN_1214950 | *Mef2c* | 1.998208168 | 0.043655 |
| ILMN_1224963 | *Syt1* | 1.966661151 | 0.002363 |
| ILMN_1232107 | *Bex2* | 1.950695969 | 0.0081 |
| ILMN_1228105 | *Sox10* | 1.944419775 | 0.024044 |
| ILMN_2874270 | *Hoxa2* | 1.925898947 | 0.012819 |
| ILMN_1236136 | *Corin* | 1.90616885 | 0.00769 |
| ILMN_2737704 | *Gja4* | 1.902270537 | 0.042647 |
| ILMN_2839027 | *Tceal6* | 1.900656218 | 0.033267 |
| ILMN_1238309 | *Rbfox1* | 1.870635477 | 0.016785 |
| ILMN_1237670 | *Entpd2* | 1.863168341 | 0.044494 |
| ILMN_2754985 | *Phlda1* | 1.784294925 | 0.003396 |
| ILMN_2593034 | *C1qtnf3* | 1.75206299 | 0.002433 |
| ILMN_2645208 | *Arhgef3* | 1.734014689 | 0.0257 |
| ILMN_2643212 | *Rprm* | 1.717329172 | 0.014218 |
| ILMN_2642417 | *Mest* | 1.708667053 | 0.00302 |
| ILMN_2709087 | *Meox2* | 1.703368731 | 0.012018 |
| ILMN_2611295 | *Dusp26* | 1.694619997 | 0.005768 |
| ILMN_2791644 | *Reep1* | 1.681422443 | 0.018352 |
| ILMN_1221750 | *Mycl* | 1.676556806 | 0.042234 |
| ILMN_2422615 | *Ebf3* | 1.670695317 | 0.030358 |
| ILMN_2769884 | *Igf1* | 1.662646425 | 0.033701 |
| ILMN_2598083 | *Hoxb2* | 1.628245785 | 0.008923 |
| ILMN_2819558 | *Bach2* | 1.616365868 | 0.013886 |
| ILMN_2612283 | *Gata2* | 1.614598008 | 0.00836 |
| ILMN_2850391 | *Meis2* | 1.608295937 | 0.004128 |
| ILMN_2592321 | *Cdh13* | 1.598061456 | 0.024196 |
| ILMN_1252636 | *Mmd2* | 1.593162998 | 0.03602 |
| ILMN_2691641 | *Gja5* | 1.58969708 | 0.029947 |
| ILMN_2878501 | *Nedd4l* | 1.58497709 | 0.00353 |
| ILMN_2659960 | *Lrba* | 1.572137018 | 0.018615 |
| ILMN_2649172 | *Zcchc18* | 1.566957872 | 0.037706 |
| ILMN_1233840 | *Kif1a* | 1.565486619 | 0.016286 |
| ILMN_2689784 | *Mcf2l* | 1.557936214 | 0.040641 |
| ILMN_2628603 | *Limch1* | 1.555375453 | 0.022327 |
| ILMN_2420465 | *Zfp703* | 1.549855455 | 0.009763 |
| ILMN_1215136 | *Scn3b* | 1.539419844 | 0.046588 |
| ILMN_2707137 | *Jade1* | 1.538280429 | 0.023179 |
| ILMN_1242617 | *Lpar4* | 1.531025047 | 0.013066 |
| ILMN_2423249 | *Tnrc6a* | 1.527158154 | 0.036159 |
| ILMN_1233507 | *Myod1* | 1.525275339 | 0.026959 |
| ILMN_1254975 | *Has2* | 1.52372243 | 0.022363 |
| ILMN_2740852 | *F2r* | 1.509834493 | 0.023387 |
| ILMN_1246069 | *Ndufab1* | 1.509355115 | 0.022274 |
| ILMN_2591158 | *Fam13c* | 1.504863313 | 0.027897 |
| ILMN_1246201 | *Cacna1h* | 1.504293959 | 0.006616 |
| ILMN_2954987 | *Mb* | 1.504286676 | 0.047308 |
| ILMN_2632092 | *Ppp1r9a* | 1.503248491 | 0.01795 |
| ILMN_1226016 | *Scx* | 1.503021628 | 0.013032 |
| ILMN_1218051 | *Iqgap2* | 1.490868237 | 0.01804 |
| ILMN_1216021 | *Ttyh1* | 1.484453979 | 0.013461 |
| ILMN_1242977 | *Hoxb5* | 1.481930526 | 0.040472 |
| ILMN_2484322 | *Ank3* | 1.480987809 | 0.046417 |
| ILMN_1216279 | *Irf6* | 1.476185622 | 0.043249 |
| ILMN_1243949 | *Dner* | 1.473155093 | 0.048663 |
| ILMN_1237186 | *Spint1* | 1.472208713 | 0.02827 |
| ILMN_2982771 | *Fgf10* | 1.471644749 | 0.014429 |
| ILMN_2622374 | *Ndn* | 1.471394528 | 0.011473 |
| ILMN_2840286 | *Megf10* | 1.470081258 | 0.043305 |
| ILMN_2494244 | *Vdac2* | 1.450817844 | 0.003231 |
| ILMN_1216662 | *Kif5c* | 1.443618929 | 0.04662 |
| ILMN_2665490 | *Litaf* | 1.442693941 | 0.036323 |
| ILMN_2773169 | *Grb7* | 1.441043044 | 0.048845 |
| ILMN_2726585 | *Nfib* | 1.439957338 | 0.008097 |
| ILMN_2677569 | *Fam155a* | 1.433748055 | 0.045587 |
| ILMN_2729197 | *Hic1* | 1.432530265 | 0.023883 |
| ILMN_1224110 | *Bst2* | 1.431113721 | 0.028579 |
| ILMN_2864170 | *Chst3* | 1.430568835 | 0.012487 |
| ILMN_3161289 | *Gnaz* | 1.426650294 | 0.001092 |
| ILMN_2636463 | *Tcf4* | 1.423011525 | 0.006662 |
| ILMN_2901409 | *Tmem255a* | 1.421709759 | 0.011745 |
| ILMN_1256408 | *Tshz2* | 1.41533208 | 0.01808 |
| ILMN_2945491 | *Myh7* | 1.41004965 | 0.033959 |
| ILMN_2585867 | *Jph1* | 1.408384753 | 0.048244 |
| ILMN_2991912 | *Rab3a* | 1.401934136 | 0.009043 |
| ILMN_1226261 | *Emc9* | 1.396178773 | 0.017244 |
| ILMN_2676052 | *Tef* | 1.395108133 | 0.024887 |
| ILMN_1230788 | *Tle1* | 1.394029536 | 0.037207 |
| ILMN_1242039 | *Med14* | 1.394002086 | 0.029596 |
| ILMN_1239294 | *Cdc42ep2* | 1.393260994 | 0.031516 |
| ILMN_1216143 | *Rnf11* | 1.389707703 | 0.010262 |
| ILMN_1244081 | *Rgs4* | 1.389089904 | 0.044573 |
| ILMN_1241890 | *Klf7* | 1.388561395 | 0.031216 |
| ILMN_2804166 | *Igsf9* | 1.382812941 | 0.026112 |
| ILMN_1247075 | *Npc1* | 1.367706056 | 0.046119 |
| ILMN_2510694 | *Atp1b1* | 1.358814969 | 0.023361 |
| ILMN_1241915 | *Notch1* | 1.357279499 | 0.039779 |
| ILMN_2693052 | *Hoxd8* | 1.347562462 | 0.042471 |
| ILMN_2670959 | *Snrnp70* | 0.741010295 | 0.046973 |
| ILMN_1217098 | *Drd4* | 0.740092565 | 0.035752 |
| ILMN_2773447 | *Ssr4* | 0.738430669 | 0.039328 |
| ILMN_1222503 | *Alg5* | 0.737438642 | 0.030161 |
| ILMN_1253600 | *Trnp1* | 0.737289966 | 0.046728 |
| ILMN_1232184 | *Ggcx* | 0.735986097 | 0.048919 |
| ILMN_1240883 | *Rnf4* | 0.735242067 | 0.038757 |
| ILMN_2894991 | *Sf1* | 0.730986636 | 0.020179 |
| ILMN_2727273 | *Swap70* | 0.730750967 | 0.042242 |
| ILMN_2910653 | *Bmp8a* | 0.726128733 | 0.028913 |
| ILMN_2697766 | *Chtf8* | 0.725468954 | 0.02554 |
| ILMN_3161263 | *Fzd5* | 0.722529409 | 0.009084 |
| ILMN_1218116 | *Bmp1* | 0.720331518 | 0.035337 |
| ILMN_1226666 | *Cnn2* | 0.720150249 | 0.030195 |
| ILMN_2838871 | *Sidt2* | 0.719880618 | 0.040848 |
| ILMN_2706232 | *Syngr2* | 0.718421867 | 0.023484 |
| ILMN_2861176 | *Calr* | 0.71811154 | 0.008906 |
| ILMN_2999818 | *Slc17a9* | 0.717124129 | 0.012689 |
| ILMN_1250947 | *Txndc5* | 0.716909596 | 0.037082 |
| ILMN_2870883 | *Yrdc* | 0.715620809 | 0.030308 |
| ILMN_1239319 | *Entpd6* | 0.714906994 | 0.035062 |
| ILMN_1259610 | *Cd276* | 0.714737034 | 0.045431 |
| ILMN_3104118 | *Pkdcc* | 0.713155275 | 0.035096 |
| ILMN_1254437 | *Slc31a1* | 0.713053811 | 0.047158 |
| ILMN_2619491 | *Arfip2* | 0.711356815 | 0.005778 |
| ILMN_2607926 | *Kdelr2* | 0.710120485 | 0.033857 |
| ILMN_1227875 | *Fkbp7* | 0.70983222 | 0.019146 |
| ILMN_3160697 | *Angel2* | 0.7097548 | 0.007783 |
| ILMN_3123473 | *Aldh18a1* | 0.708699877 | 0.032969 |
| ILMN_2975345 | *Cdo1* | 0.707762052 | 0.019635 |
| ILMN_2705966 | *Sec31a* | 0.705784589 | 0.009289 |
| ILMN_2907499 | *Tatdn2* | 0.702687266 | 0.016361 |
| ILMN_2596230 | *Mlec* | 0.702357083 | 0.045811 |
| ILMN_2525799 | *H2afy2* | 0.701474466 | 0.017506 |
| ILMN_2878274 | *Copz2* | 0.700071778 | 0.011932 |
| ILMN_2592093 | *Ift20* | 0.698323385 | 0.032897 |
| ILMN_2883952 | *Ydjc* | 0.697585295 | 0.044423 |
| ILMN_1253468 | *Col24a1* | 0.69624929 | 0.018498 |
| ILMN_1255177 | *Pdia5* | 0.694181866 | 0.010494 |
| ILMN_2441909 | *Tbc1d2b* | 0.692092458 | 0.024872 |
| ILMN_2633350 | *Mfap4* | 0.690784412 | 0.048961 |
| ILMN_2776764 | *Chmp4b* | 0.688801596 | 0.036166 |
| ILMN_3114641 | *Pik3r1* | 0.685402961 | 0.025606 |
| ILMN_2687974 | *Spryd3* | 0.681994683 | 0.031295 |
| ILMN_1243690 | *Ptbp1* | 0.679749985 | 0.046888 |
| ILMN_1237572 | *Aard* | 0.679195611 | 0.019524 |
| ILMN_1225332 | *Tmsb10* | 0.678687223 | 0.037105 |
| ILMN_1254634 | *Pxylp1* | 0.678513498 | 0.023908 |
| ILMN_2650732 | *Taf15* | 0.67724641 | 0.004843 |
| ILMN_2625687 | *Galnt1* | 0.674891215 | 0.01798 |
| ILMN_2669793 | *Ccnd1* | 0.667791937 | 0.030053 |
| ILMN_1256639 | *Cd247* | 0.667459598 | 0.019558 |
| ILMN_2739128 | *Tmem214* | 0.666470338 | 0.006581 |
| ILMN_3147944 | *Ap2b1* | 0.665962033 | 0.020668 |
| ILMN_2996732 | *Slc39a13* | 0.661550416 | 0.035383 |
| ILMN_2445165 | *Vdr* | 0.661506116 | 0.034486 |
| ILMN_2980226 | *Mmp16* | 0.661287133 | 0.036195 |
| ILMN_2705235 | *Prex1* | 0.659622241 | 0.038401 |
| ILMN_2523316 | *Unc5b* | 0.65623598 | 0.040307 |
| ILMN_2956942 | *Prkcdbp* | 0.654849679 | 0.02535 |
| ILMN_2863903 | *Rnf25* | 0.65138202 | 0.03333 |
| ILMN_2664155 | *Pycr1* | 0.650670928 | 0.001349 |
| ILMN_2626389 | *Nomo1* | 0.649987471 | 0.044922 |
| ILMN_1238597 | *Omd* | 0.649819577 | 0.024628 |
| ILMN_2927638 | *Mxra8* | 0.64955644 | 0.034882 |
| ILMN_2637714 | *Rasa3* | 0.647561568 | 0.03815 |
| ILMN_2651190 | *Gosr2* | 0.647207251 | 0.036614 |
| ILMN_1227836 | *Arpc1b* | 0.645710189 | 0.022858 |
| ILMN_1233809 | *Cmtm7* | 0.645265699 | 0.027753 |
| ILMN_2656871 | *Tmem97* | 0.638647023 | 0.045717 |
| ILMN_2612079 | *Rrbp1* | 0.637142452 | 0.036169 |
| ILMN_2729458 | *Idh1* | 0.633453751 | 0.046605 |
| ILMN_1241903 | *Klf4* | 0.630829339 | 0.022885 |
| ILMN_2694381 | *Dnajc7* | 0.630767397 | 0.031437 |
| ILMN_2502471 | *Fam46a* | 0.630598279 | 0.008364 |
| ILMN_3162476 | *Sec61a1* | 0.628087475 | 0.042002 |
| ILMN_2526875 | *Gm4788* | 0.627661826 | 0.008256 |
| ILMN_2766894 | *Enpp6* | 0.624437443 | 0.015286 |
| ILMN_2734212 | *Cd1d1* | 0.618892797 | 0.041394 |
| ILMN_1237364 | *Mmp23* | 0.613689959 | 0.002268 |
| ILMN_2711461 | *Tgfb1* | 0.60821672 | 0.035625 |
| ILMN_2841289 | *Tnfaip2* | 0.605843154 | 0.02394 |
| ILMN_1238644 | *Marveld1* | 0.603050764 | 0.031044 |
| ILMN_3059326 | *Sparc* | 0.600064723 | 0.041298 |
| ILMN_2605645 | *Gsn* | 0.596221931 | 0.003157 |
| ILMN_1215085 | *Fkbp10* | 0.589322245 | 0.021939 |
| ILMN_1233455 | *Olfml3* | 0.587541837 | 0.027843 |
| ILMN_2884610 | *Ppapdc1b* | 0.584689662 | 0.002354 |
| ILMN_2760450 | *Tfap2b* | 0.582197709 | 0.001532 |
| ILMN_1253806 | *Col1a2* | 0.580382844 | 0.041023 |
| ILMN_2595260 | *Creb3l1* | 0.571871535 | 0.012618 |
| ILMN_2872058 | *Ctsh* | 0.565197601 | 0.041521 |
| ILMN_1250752 | *Kif23* | 0.560261413 | 0.018557 |
| ILMN_1253062 | *Insc* | 0.55776197 | 0.030568 |
| ILMN_2680387 | *Cpz* | 0.554742894 | 0.032896 |
| ILMN_1237629 | *Cntn2* | 0.553950218 | 0.019235 |
| ILMN_1234747 | *Adamtsl5* | 0.54502699 | 0.014085 |
| ILMN_1215849 | *Dpp8* | 0.54310122 | 0.045298 |
| ILMN_1258906 | *P3h1* | 0.542386837 | 0.014924 |
| ILMN_2637094 | *Pcsk6* | 0.536630938 | 0.023969 |
| ILMN_2698728 | *Srpx2* | 0.529034415 | 0.011447 |
| ILMN_2650356 | *Pth1r* | 0.521413397 | 0.043248 |
| ILMN_1246173 | *Msx1* | 0.511250356 | 0.038435 |
| ILMN_1227663 | *Sgms2* | 0.493771734 | 0.030106 |
| ILMN_1214899 | *Phospho1* | 0.417487421 | 0.043618 |
| ILMN_2661287 | *Alpl* | 0.417109456 | 0.038242 |
| ILMN_2669062 | *Pi16* | 0.408568983 | 0.021227 |

Fold change is calculated by arithmetic mean in linear scale. Fold change > 1 (up-regulated gene), fold change ˂ 1 (down-regulated gene). Genes in yellow studied in this manuscript.
